# Atypical residency of short-beaked common dolphins (*Delphinus delphis*) to a shallow, urbanized embayment in south-eastern Australia

**DOI:** 10.1098/rsos.160478

**Published:** 2016-09-28

**Authors:** Suzanne Mason, Chandra Salgado Kent, David Donnelly, Jeffrey Weir, Kerstin Bilgmann

**Affiliations:** 1 The Dolphin Research Institute (DRI), PO Box 77, Hastings, Victoria 3915, Australia; 2 Centre of Marine Science and Technology (CMST), Curtin University, GPO Box U1987, Perth, Western Australia 6845, Australia; 3 Department of Biological Sciences, Macquarie University, Sydney, New South Wales 2109, Australia

**Keywords:** residency, inshore community, female philopatry

## Abstract

Short-beaked common dolphins (*Delphinus delphis*) are typically considered highly mobile, offshore delphinids. This study assessed the residency of a small community of short-beaked common dolphins in the shallow, urbanized Port Phillip Bay, south-eastern Australia. The ability to identify common dolphins by their dorsal fin markings and coloration using photo-identification was also investigated. Systematic and non-systematic boat surveys were undertaken between 2007 and 2014. Results showed that 13 adult common dolphins and their offspring inhabit Port Phillip Bay, of which 10 adults exhibit residency to the bay. The majority of these adults are reproductively active females, suggesting that female philopatry may occur in the community. Systematic surveys conducted between 2012 and 2014 revealed that the dolphins were found in a median water depth of 16 m and median distance of 2.2 km from the coast. The shallow, urbanized habitat of this resident common dolphin community is atypical for this species. As a result, these common dolphins face threats usually associated with inshore bottlenose dolphin communities. We suggest that the Port Phillip Bay common dolphin community is considered and managed separate to those outside the embayment and offshore to ensure the community's long-term viability and residency in the bay.

## 1. Introduction

Residency in delphinids is known to occur in geographical locations in which resources such as prey are available regularly and predictably [1]. Thus, delphinids spend less energy searching for key resources and can invest more energy in reproduction [2]. In some cases, these geographical locations are close to dense human populations and coastal development. Inevitably, delphinids that reside close to human populations have an increased risk of exposure to anthropogenic threats. Potential impacts from human activities include a reduced prey availability due to over-fishing [3,4], marine debris entanglements [5,6], boat-strike from recreational boat traffic (e.g. [7–9]), acoustic masking of communications from underwater noise (e.g. [10,11]), PCB and organochloride contamination (e.g. [12–14]), bioaccumulation of heavy metals such as mercury [15], and potential increased risk of disease from pollution and increased stress [16,17]. These anthropogenic impacts can affect the health, survival and reproductive success of individuals and therefore the long-term existence of resident delphinid communities in urbanized regions, in particular when communities are small.

A range of delphinid species have been reported to be resident in localized geographical locations, including killer whales (*Orcinus orca*) in British Columbia, Canada and Washington state, USA [18], Hector's dolphins (*Cephalorhynchus hectori*) in Porpoise Bay, New Zealand [19], Indo-Pacific humpback dolphins (*Sousa chineses*) in waters off Hong Kong [20] and Atlantic spotted dolphins (*Stenella frontalis*) in the Bahamas [21]. For the widely researched bottlenose dolphin (*Tursiops* spp.), residency has been reported in several geographical locations both in the southern and northern hemispheres, e.g. common bottlenose dolphins (*Tursiops truncatus*) in Sarasota Bay, USA [22], the Shannon Estuary, Ireland [23] and the Moray Firth, Scotland [24], bottlenose dolphins (*Tursiops* sp*.*) in Shark Bay, Australia [25,26], and Indo-Pacific bottlenose dolphins (*Tursiops aduncus*) in Port Stephens and Jervis Bay, New South Wales, Australia [27], the Swan-Canning River, Western Australia [28] and the Richmond and Clarence Rivers, New South Wales, Australia [29]. Likewise, southern Australian coastal bottlenose dolphin communities are resident to both the Gippsland Lakes [30] and Port Phillip Bay [31], Victoria and to several regions along the coast of South Australia including the Adelaide metropolitan area in Gulf St Vincent [32,33]. Southern Australian coastal bottlenose dolphins have recently been described as a new species (Burrunan dolphin: *Tursiops australis*) [30,34–36]. The validity of this species has not yet been recognized by the wider scientific community [37,38]. We therefore refer to the bottlenose dolphins in Port Phillip Bay as coastal southern Australian bottlenose dolphins (*Tursiops* cf*. australis*). While residency has been reported for many delphinids, the residency of short-beaked common dolphins (*Delphinus delphis*) to a shallow, urbanized embayment is atypical.

Short-beaked common dolphins, hereafter referred to as common dolphins, typically inhabit open ocean environments [39] or neritic waters [40–43] and are often found in regions with complex bathymetry and high productivity [44,45]. As exceptionally mobile marine predators, common dolphins have the ability to migrate over large distances in search of prey [46] and in some regions of the world they travel in groups ranging from 10 to over 10 000 individuals [39,47]. Even though common dolphins are a ubiquitous species, residency in urbanized marine environments is rarely reported for this species [48].

Common dolphins in Australian waters are confirmed to be short-beaked common dolphins (*D. delphis*) [41–43,49]. Fine-scale genetic structuring of common dolphins along the southern Australian coast indicates that higher levels of site fidelity may be found for this species off southern Australia [43] than in other regions around the world where common dolphins show little genetic structuring (e.g. [50]). For example, in southern Australia, common dolphins have been regularly sighted in lower Gulf St Vincent, South Australia [51,52]. Whether the common dolphins are year-round residents to lower Gulf St Vincent, or only seasonal or occasional visitors to the gulf, is currently unknown. Common dolphins are also regularly seen in Port Phillip Bay, Victoria, along the south-eastern coast of Australia. Whether these animals are resident to Port Phillip Bay was unknown prior to this study presented here.

Port Phillip Bay is an urbanized, shallow, semi-enclosed embayment, a habitat that is typically associated with bottlenose dolphins (e.g. [22,23,53,54]) but not common dolphins. Here, we investigate whether common dolphins in Port Phillip Bay, in south-eastern Australia, are resident to this embayment. We show that the dorsal fins of adult common dolphins are distinctive enough to reliably identify these individuals in the bay. Photo-identification has been widely used for delphinids, in particular bottlenose dolphins (e.g. [55,56]) but has only occasionally been used for common dolphins [57]. Lastly, we also investigate common dolphin distribution in the south-eastern part of the bay and relate this to distance from shore and water depth. Clarifying the residency status, distribution and individual identification of common dolphins in Port Phillip Bay will provide information directly applicable to future management of these dolphins in this heavily urbanized embayment, where dolphins are regularly exposed to human activities.

## 2. Material and methods

### 2.1. Study site

**Figure 1.**
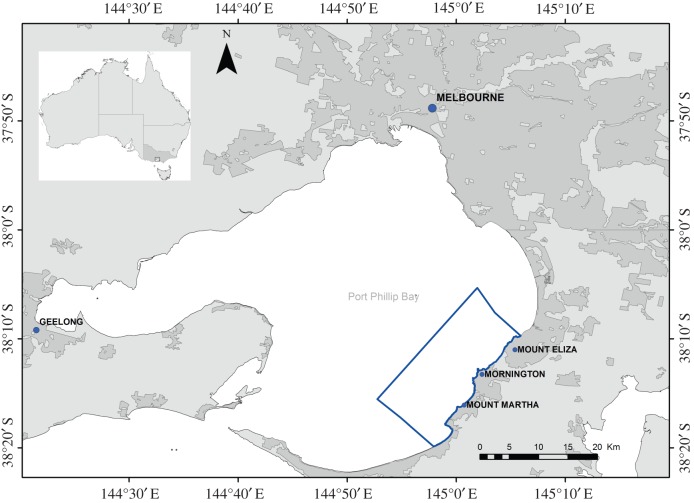
Port Phillip Bay, Victoria, and its location along the southern Australian coastline. The blue line represents the outer margins of the study area. Darker grey areas represent the urbanized regions of Melbourne, Greater Melbourne (suburbs) and Geelong that surround Port Phillip Bay.

Port Phillip Bay (38°09′ S, 144°52′ E), also referred to as Port Phillip, in the eastern part of southern Australia, is a shallow, semi-enclosed marine embayment of approximately 1930 km^2^ [58] (figure 1). Almost 50% of the bay is less than 8 m deep, while the deepest section in the centre reaches 24 m [58]. Two cities are located on the Port Phillip Bay coast: Melbourne, with a population of 4.44 million people, and Geelong, with a population of 260 000 people [59]. Port Phillip Bay is circular in shape, with a gently sloping underwater topography on the western coast and much steeper benthic gradients along the eastern and southern coast [58]. The higher cliffs and more complex underwater topography of the eastern coast are a result of the Selwyn Fault and its subsequent geological activity. The fault line runs along the eastern coastline (the study's survey area) and south to McCrae [60,61]. Port Phillip Bay is connected to Bass Strait via a 3.2 km wide entrance, located in the southern end of the bay [62,63]. Ocean swells dissipate as they move through the bay's entrance and consequently, with a lack of swell, wave action beyond the entrance is dictated by the wind. These environmental conditions, combined with the bay's shape and shallow depth, result in the Port Phillip embayment being similar to a marine lake [63].

### 2.2. Survey effort

Vessel-based surveys were completed using a 6.5 m Swordfish Savage vessel (‘*Delphindae*’) powered by a 135 hp outboard engine or a 5.5 m Gemini rigid hull inflatable boat (‘*Krillseeker*’) with a 115 hp outboard motor. Non-systematic surveys were undertaken between May 2007 and December 2011, and systematic surveys from July 2012 to July 2014. Off-effort sightings of dolphins between July 2012 and July 2014, i.e. while not on transect, and when travelling to and from start and end points of the survey route, were included in the non-systematic survey dataset. Here, we combine data collected from different survey types to assess residency of common dolphins in Port Phillip Bay. Survey design, coverage probability and effort varied among survey types. Data to correct for effort were not available for the majority of the surveys, hence no effort-based corrections were applied in this study. Unequal coverage probability was considered during the interpretation of the results.

### 2.3. Non-systematic: random survey routes

**Figure 2.**
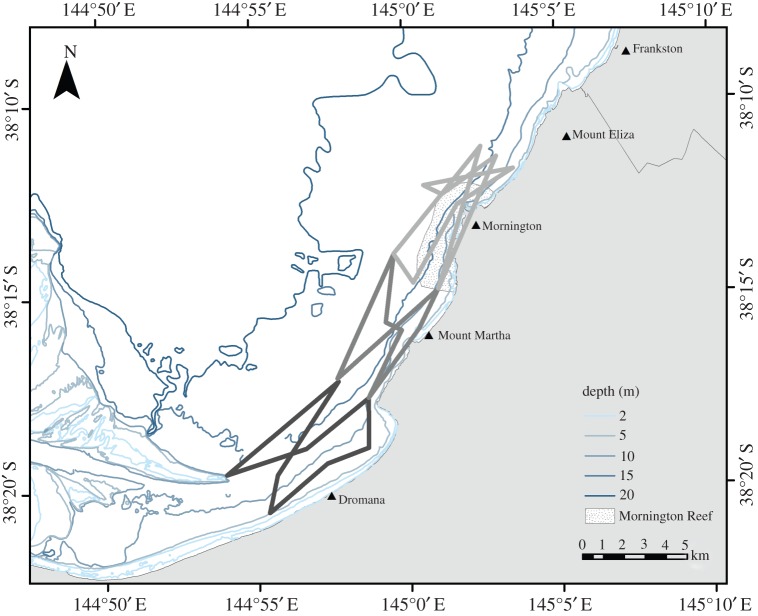
Examples of non-systematic, random transect line surveys along the Mornington (light grey lines), Mount Martha (dark grey lines) and Dromana (black lines) coasts. Planned survey routes ran over the coast, but actual survey routes deviated and followed the coastline as close as practical.

Random-line surveys were run between May and August 2007. Using a random number chart, the order of six to eight waypoints and lines of travel between them formed the survey route. The waypoints were positioned at the corners of the survey area, midway along the outer edge and in the centre (figure 2). The route was prepared in PC Planner v. 11.02 [64] and transferred to the vessel's chartplotter at the commencement of each survey. This survey method, while random, did not allow for any point within the survey area to have equal sampling probability. Thus, the random-line surveys did not fully meet the assumptions of conventional distance sampling [65].

### 2.4. Non-systematic: haphazard survey routes

Haphazard survey routes were run between June 2008 and May 2012. The research vessel was launched where common dolphins were historically sighted and a decision was made to survey either north or south of the launch site after visibility and sea state were considered. The vessel route usually incorporated an inshore track that paralleled the coast and an equivalent track further offshore (design not presented here).

### 2.5. Systematic survey routes

**Figure 3.**
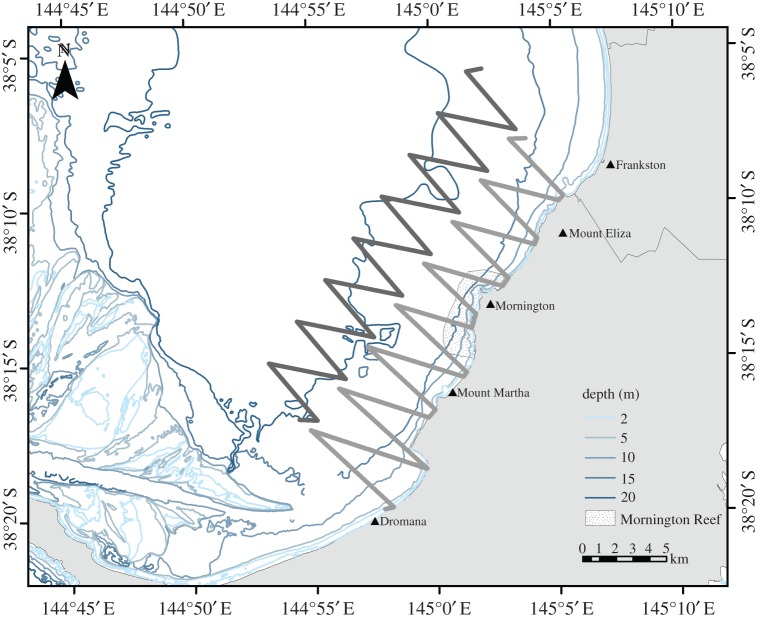
Example of systematic line surveys conducted both inshore (light grey) and offshore (dark grey) between Mount Eliza and Dromana.

Systematic surveys covering an area of 213 km^2^ were run from July 2012 until July 2014 and were pre-planned in DISTANCE 6.0 [66]. Surveys were specifically designed to provide homogeneous coverage probability of the survey areas. An equally spaced zigzag design was selected to reduce the time required to travel from one transect line to the next (figure 3). Survey routes incorporated at least 15 transect lines ran approximately perpendicular to the coast and had starting points randomly generated in DISTANCE. The survey area was divided into inshore (up to 5 km from shore) and offshore (5–10 km from shore). Inshore systematic surveys covered the same general area of the earlier non-systematic random and haphazard surveys. The offshore survey routes were designed to extend beyond the non-systematic survey routes to investigate common dolphin occurrence further from the coast. Total survey track length, for each of the inshore and offshore surveys, ranged between 65 and 85 km. Surveys were run in closing mode, during which the vessel left the transect line to ‘close in’ on the dolphins to obtain detailed observations [67].

### 2.6. All survey types

All surveys were undertaken in Beaufort Sea State less than or equal to 3, with the research vessel travelling at speeds between 12 and 15 knots. Upon sighting dolphins, initial behaviour, approximate group size, the presence of calves and the travel direction of the group were recorded. Once the pre-approach observations were complete, the research vessel approached the dolphins to collect dorsal fin-identification images. Individuals were considered to be a group when they were within 10 m of each other [25] and exhibited the same behaviours and coordinated movement in the same general direction [68]. Where the same group of dolphins was re-sighted in one day, only the first sighting was used in the analysis. Once all photo-identification was completed, the vessel returned to the location on the transect line where it had left and continued the survey.

### 2.7. Photo-identification and gender determination

Dorsal fin-identification images were captured using a Canon 30D or 50D camera with L series 70–200 mm lenses. For identification of individuals, both the accumulated unique nicks and notches on the trailing edge of the dolphin's dorsal fin [69,70] and fin coloration [57] were used. The gender of individuals was obtained opportunistically. Common dolphins with a postanal hump were identified from photographs as mature males [71,72]. Females were identified through the presence of an accompanying calf during more than two surveys, and/or through the presence of mammary slits opportunistically photographed when inverted. The common dolphin's size and coloration as described by Jefferson [39] were used to determine its life stage; stages were defined as calf, sub-adult and adult. Calves had a reduced body size of 1/3 to 1/2 the size of adults in the group with a body coloration generally muted and faint borders where differing colorations met. Sub-adults were of a slightly smaller size than adults and coloration, although developed, was fainter than in adults. Adults showed expected size ranges of an adult and had fully developed bold body coloration.

## 3. Data analysis

### 3.1. Sighting rates and residency status

For this study, individuals were considered residents if they were recorded in Port Phillip Bay for more than 50% of the seasons during the study period. This was adopted from Rosel *et al.* [73], where individuals were considered residents when they spent more than 50% of their time in a specific area in a given year. In this study, seasons were based on the austral seasons: summer (December to February), autumn (March to May), winter (June to August) and spring (September to November).

### 3.2. Photo analysis

Dolphins were identified both while in the field and post-survey from images taken during close approaches. Images were assessed for clarity, contrast, angle to the camera, full fin in image frame and distance to the camera [74], with each criterion weighted based on its importance [75]. Images of poor quality were not included in the analysis. Distinctiveness of each dorsal fin was determined based on fin features as described by Urian *et al.* [75]. Distinguishing variations in dorsal fin coloration patterns, which included darker coloured patches and mottling, were also considered for each individual. These differences were compared across a variety of lighting conditions in which individuals were photographed, to ensure that they were actual identifiable differences and not just the products of variations of lighting on the day of survey. Fin photographs of sub-adult common dolphins and calves were also taken although not included in the analysis due to the lack of distinguishing features on their dorsal fins [76].

### 3.3. Distribution

ArcMap 10.2 [77] was used to map the locations of all initial dolphin group sightings made during systematic and non-systematic surveys. Depth data were obtained from the Australian Hydrographic Service [78] and converted from S.57 format to a shapefile for use in ArcMap. Raster layers were created for both water depth and Euclidean distance from shore. Depth and distance from shore were extracted from the raster layers according to each location point where dolphins had been initially sighted. The point data were then exported into an Excel spreadsheet and imported into the computational software R [79] run through Rstudio v. 0.99.441 © 2009–2015, RStudio Inc. for statistical analysis and graphical output.

## 4. Results

Forty-eight surveys, including both non-systematic and systematic survey routes, were undertaken along the eastern coast of Port Phillip Bay between 2007 and 2014 and used to determine common dolphin residency. Common dolphins were encountered during 85% of the surveys and 60 initial sightings of common dolphin groups were recorded across the survey period (table 1).

**Table 1.** Details of survey effort and number of short-beaked common dolphin (*Delphinus delphis*) groups sighted for each survey type.

surveys	survey effort (h)	time with dolphins (h)	distance covered	groups sighted
non-systematic (random line *n* = 6, haphazard *n* = 21, off-effort systematic *n* = 8)	164.80	28.67	not recorded	46
systematic (inshore *n* = 13, offshore *n* = 8)	74.37	7.25	1628.7 km	14
total	239.17	35.92		60

A total of 13 individual adult common dolphins were identified from 4055 photo-identification images taken during the surveys. No observed adults were unmarked or unidentifiable. In 2007, only seven adult common dolphins were sighted. Between 2008 and 2014, 12 adults were sighted regularly (table 2). Of the 13 identified adult individuals, 10 were identified as female, one (ID 9001) as a male, and two were of unknown gender (table 3). In 2012, one dolphin (ID 10002) was identified for the first time, while another (ID 10101; gender unknown) had not been sighted during surveys since late 2012. Dorsal fin markings, shape and coloration showed clear differences between these two animals and thus it could not have been the same animal obtaining additional marks to its dorsal fin. Fourteen calves were born in the Port Phillip Bay common dolphin community between 2007 and 2014, of which the majority were born during the second half of the study period. As this study focused on the adult dolphins in the community, survivorship of the calves was not estimated. The common dolphin community is considered to be small, based on the numbers of adult common dolphins, calves born during the study period, and sub-adult individuals sighted in groups separate to the adults and calf groups. In total, the Port Phillip Bay common dolphin community is estimated to comprise approximately 30 individuals.

**Table 2.** Sightings of individual adult short-beaked common dolphin (*Delphinus delphis*) during 48 surveys in Port Phillip Bay, south-eastern Australia between May 2007 and July 2014. Green shading indicates an individual sighted during a non-systematic survey, dark blue shading during an inshore systematic survey and light blue during an offshore systematic survey. A black outline surrounding a green shaded box indicates that the sighting was made while off-effort during a systematic survey, hence the sighting was included in the non-systematic survey data. Where shading is absent for a survey column, no common dolphins were encountered during the survey.

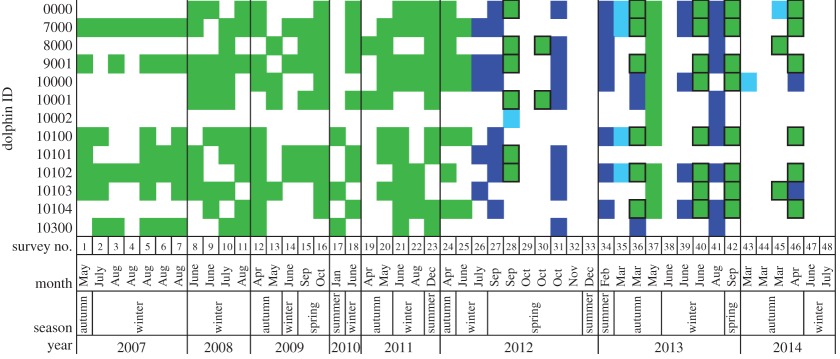

**Table 3.** Sighting rates of adult short-beaked common dolphin (*Delphinus delphis*) observed along the south-eastern coast of Port Phillip Bay during 21 seasons between 2007 and 2014. Sighting rates are based on a definition of residency adapted from Rosel *et al.* [73] with the number of seasons sighted based on austral seasons. Dolphins with a sighting rate more than or equal to 50% were considered resident to Port Phillip Bay and are indicated in italics.

ID no.	dolphin	year first sighted	no. seasons observed	seasons observed	percentage of seasons sighted across survey period
0000	V-Nick	2008	13	Sum, Aut, Wint, Spr	*61.9*
7000	Esther	2007	18	Sum, Aut, Wint, Spr	*85.7*
8000	Almost Clean Fin	2008	12	Sum, Aut, Wint, Spr	*57.1*
9001	Tall Fin	2007	18	Sum, Aut, Wint, Spr	*85.7*
10000	Round Mid Notch	2008	14	Sum, Aut, Wint, Spr	*66.7*
10001	Square Notch	2008	9	Sum, Aut, Wint, Spr	42.9
10002	Funky Fin	2012	3	Aut, Wint, Spr	14.3
10100	Triple Nick	2007	15	Sum, Aut, Wint, Spr	*71.4*
10101	Spot	2007	11	Sum, Aut, Wint, Spr	*52.4*
10102	Ragged Fin	2007	15	Sum, Aut, Wint, Spr	*71.4*
10103	Barrett	2007	14	Sum, Aut, Wint, Spr	*66.7*
10104	Poke	2008	14	Sum, Aut, Wint, Spr	*66.7*
10300	Scroll	2007	9	Sum, Aut, Wint, Spr	42.9

### 4.1. Re-sighting rates and site fidelity

Ten of the adult common dolphins from the community had sighting rates more than 50%, i.e. 52.4%–85.7%, indicating their residency to Port Phillip Bay (table 3). The remaining three adults had sighting rates of 14.3%, 42.9% and 42.9%, respectively (table 3).

### 4.2. Photo-identification and fin distinctiveness

All adult common dolphins photographed between 2007 and 2014 in Port Phillip Bay had either distinct, moderate or marginally distinct dorsal fins with varying coloration patterns and were therefore individually identified and included in a long-term dorsal fin catalogue.

**Figure 4.**
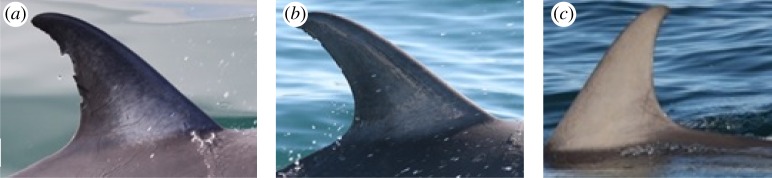
Distinctiveness in the edge of the dorsal fin of adult short-beaked common dolphin (*Delphinus delphis*) dorsal fins from Port Phillip Bay, south-eastern Australia, based on Urian *et al.* [75]. (*a*) ‘Very distinct fin’ (D1), dolphin 10102; (*b*) ‘Moderately distinct fin’ (D2), dolphin 10103; and (*c*) ‘Marginally distinct fin’ (DM), dolphin 9001.

Damage to adult common dolphin dorsal fins, mainly the trailing edge, resulted in varying levels of distinctiveness (table 4 and figure 4). Three of the adult dolphins had very distinct dorsal fins (D1), eight had one or two features on their dorsal trailing edge (D2), and the dorsal fins of two dolphins had marginally distinct features (DM). No adult common dolphin in the community had a dorsal fin without distinctive markings (ND). All individuals with non-distinct dorsal fins were calves and sub-adults.

**Table 4.** List of identified adult short-beaked common dolphin (*Delphinus delphis*) in Port Phillip Bay, south-eastern Australia, including dolphin ID number, name, gender, age, years in which calves were born and fin distinctiveness. Fin distinctiveness was determined using categories defined by Urian *et al.* [75] for bottlenose dolphins. Categories were *very distinctive* (D1) fins with multiple features; *moderately distinctive* (D2), one major feature or two features; *marginally distinctive* (DM), markings, pattern, leading and trailing edge features of dorsal fin provide little information; and *not distinctive* (ND), markings, pattern, leading and trailing edge features of dorsal fin provide no information.

ID no.	dolphin name	gender	age class	years calves were born	fin distinctiveness category
0000	V-Nick	female	adult	2009, 2013	D2
7000	Esther	female	adult	2007, 2010, 2013	D2
8000	Almost Clean Fin	female	adult	2012, 2014	DM
9001	Tall Fin	male	adult		DM
10000	Round Mid Notch	female	adult	2009, 2011, 2013	D2
10001	Square Notch	female	adult	2012	D2
10002	Funky Fin	female	adult		D1
10100	Triple Nick	unknown	adult		D1
10101	Spot	unknown	adult		D2
10102	Ragged Fin	female	adult		D1
10103	Barrett	female	adult	2012,	D2
10104	Poke	female	adult	2009, 2012	D2
10300	Scroll	female	adult		D2

**Figure 5.**
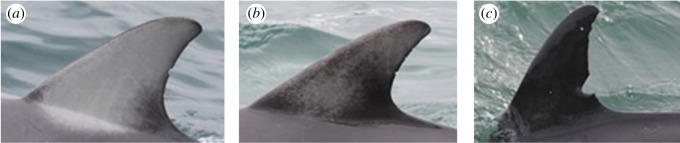
Differences in the coloration pattern of adult short-beaked common dolphin (*Delphinus delphis*) dorsal fins from Port Phillip Bay, south-eastern Australia. (*a*) Pale common dolphin dorsal fin, dolphin 10000; (*b*) Intermediate coloration, dolphin 10100; and (*c*) almost black common dolphin dorsal fin, dolphin 10002.

**Figure 6.**
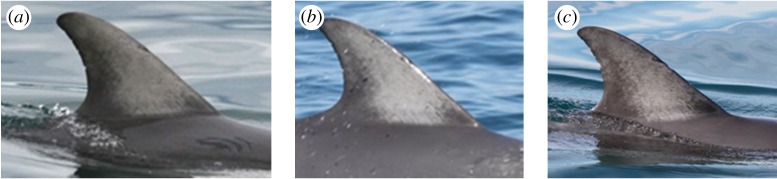
An example of stable coloration pattern of an adult short-beaked common dolphin (*Delphinus delphis*) dorsal fin from Port Phillip Bay, south-eastern Australia, over time. Dolphin 10100 photographed in (*a*) 2007, (*b*) 2010 and (*c*) 2013.

The coloration of individual dorsal fins ranged from pale (figure 5*a*) to uniformly dark (figure 5*c*), with some individuals showing an intermediate coloration (figure 5*b*). Fin coloration pattern of the adult common dolphins appeared to remain stable over time and was used to identify individuals both in the field and from images (figure 6).

### 4.3. Sighting locations

**Figure 7.**
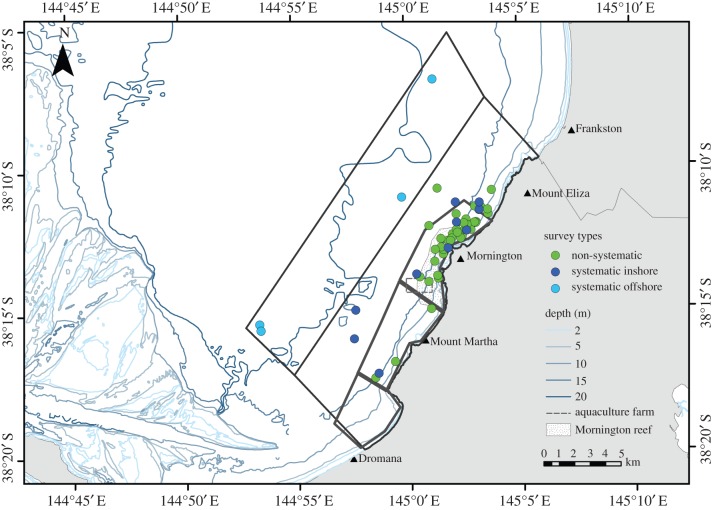
Locations of initial sightings of adult short-beaked common dolphins (*Delphinus delphis*) in Port Phillip Bay, south-eastern Australia, encountered during systematic and non-systematic surveys between May 2007 and July 2014. Green circles represent initial sightings of common dolphin groups recorded during non-systematic surveys (*n* = 39), dark blue circles those made during inshore systematic surveys (*n* = 10) and light blue circles those during offshore systematic surveys (*n* = 4). The light grey lines enclose the areas of the inshore (*n* = 13) and offshore (*n* = 8) systematic surveys. Dark grey lines enclose the areas traversed during the non-systematic random-line surveys; each of the three sections was surveyed six times. Random-line and haphazard survey routes were conducted within the inshore systematic survey area.

**Figure 8.**
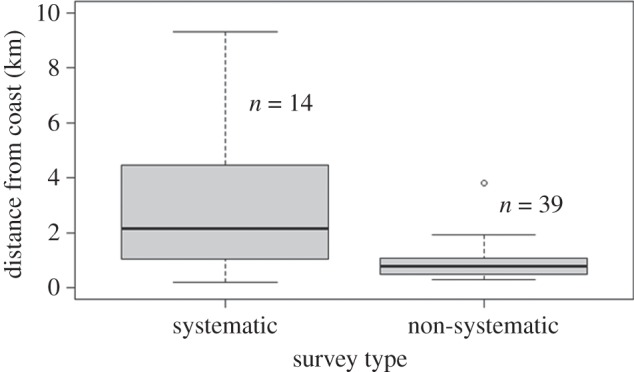
Distribution of distances from shore of resident adult short-beaked common dolphins (*Delphinus delphis*) encountered in Port Phillip Bay, south-eastern Australia, during systematic and non-systematic surveys. The dark line in the boxplots represent the median distance from shore that common dolphins were encountered. The box represents distances from the coast falling within the 25th and 75th percentiles, while the upper and lower ‘whiskers' represent the furthest and closest distances (respectively) greater than or equal to the interquartile range that dolphins were observed from shore. The black circle is an outlier and represents distance observations that lie beyond upper or lower interquartile marks.

Common dolphins in Port Phillip Bay were generally seen between Mount Eliza and Mount Martha, an area with distinct underwater topography caused by the formation of the Selwyn Fault and its subsequent geological activity (figure 7) [60,61]. Distance (Euclidean) of dolphin sightings from shore was calculated for systematic and non-systematic surveys (figure 8). During systematic surveys, the distance from shore for common dolphin groups ranged from 0.2 km to 9.3 km with a median distance of 2.2 km. GPS data were not available for four non-systematic surveys, hence the distance from shore for seven common dolphin sightings could not be calculated. During non-systematic surveys common dolphins were encountered between 0.3 and 3.8 km from shore with a median distance of 781 m. The non-systematic survey distances of up to 3.8 km from shore represented 64% of encounters during systematic surveys, indicating that the core range of common dolphins within the survey area may lie within 3.8 km from shore. The remaining 36% of distances measured during systematic surveys were beyond 3.8 km from shore.

**Figure 9.**
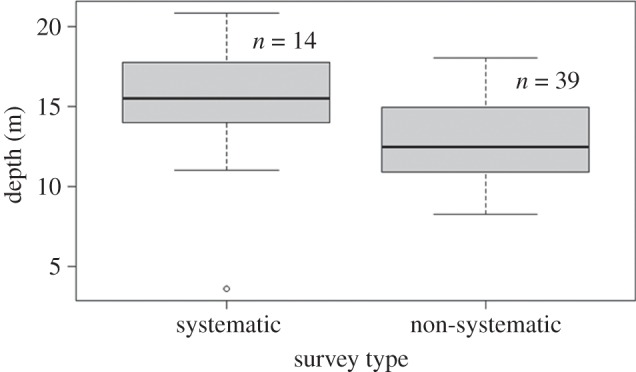
Water depths in which resident adult short-beaked common dolphins (*Delphinus delphis*) were sighted in Port Phillip Bay, south-eastern Australia, during systematic and non-systematic surveys. The dark lines in the boxplots represent the median depths from shore that common dolphins were encountered. The boxplot signifies the interquartile range of depths. The boxes represent depths from the coast falling between the 25th and 75th percentiles, while the upper and lower ‘whiskers’ represent the deepest and shallowest depths the dolphins were observed in, respectively. The black circle represents an outlier sighting.

Water depths in which common dolphins were encountered were plotted for systematic and non-systematic surveys (figure 9). Systematic surveys indicated that common dolphins were found in depths ranging from 4 to 21 m with a median depth of 16 m. GPS data were not available for four non-systematic surveys. Hence, the depth for seven common dolphin sightings could not be calculated. Non-systematic surveys indicated common dolphins were encountered in depths ranging from 8 to 18 m with a median depth of 12 m. The non-systematic survey depths of up to 18 m represented 79% of the depths in which the common dolphins were encountered during systematic surveys.

## 5. Discussion

This study revealed that a total of 10 adult common dolphins are resident to this embayment. Both residencies to a bay and small community size are atypical for this generally gregarious neritic and offshore species. The number of adult common dolphins identified and re-sighted in Port Phillip Bay increased from seven in 2007 to 12 in 2008, of which three were identified as occasional visitors. Historically, two common dolphins were opportunistically sighted in 1995 in the southern region of Port Phillip Bay [80]. As no common dolphin surveys were conducted during this time, the number of individuals that were regularly found in the bay in these early years remains unknown. However, common dolphins were thought to be rare or casual visitors to the embayment [80,81]. In this study, 10 adult common dolphins had a sighing rate greater than 50% between 2007 and 2014, hence were considered residents to the bay. The majority of adult dolphins were first sighted in 2007 and 2008, suggesting that the community consists of a relatively stable number of individuals. After 2008, the only change of adults to the community was one individual (dolphin 10002) that was first identified in the community in 2012, and another (dolphin 10101) that was not re-sighted after late 2012; both individuals were distinct in their dorsal fin markings. Thus, little immigration and emigration of adult dolphins has occurred over the study period. Altogether, 10 of the 13 adult common dolphins observed in the bay during this study display residency to the south-eastern region of the Bay, an area with distinct underwater topography. When including unmarked calves and sub-adult animals, the Port Phillip community is estimated to consist of around 30 common dolphins. Ultimately, 13 adult common dolphins, 10 of which are resident, is a remarkably low number of dolphins that form a community in this embayment, which is atypical for this generally gregarious neritic and offshore species.

Residency in dolphins generally occurs when resources are spatially and temporally predictable [1]. Although the Port Phillip embayment is much shallower than the habitat in which common dolphins are typically found, the eastern region of Port Phillip Bay has a distinct bottom topography and is likely to be productive enough to sustain the small community and facilitate residency in the area. Common dolphins prey mostly on schooling fish species [82–84] and are often observed feeding cooperatively [85]. In South Australian waters, stomach contents of beach cast and bycaught common dolphins revealed that anchovies (*Engraulis australis*) were one of the most consumed prey (41.0%) [83]. Port Phillip Bay supports the largest of the commercial anchovy fisheries in Victorian waters [86]. Furthermore, the anchovies that occur in Port Phillip Bay are an important prey species for the little penguin (*Eudyptula minor*) [87]. Thus, the bay is an important foraging ground for the Phillip Island little penguin colony during winter when the abundance of available prey in local Bass Strait waters outside of the bay is thought to be reduced [88,89]. It is therefore likely that anchovies are also one of the target prey species for common dolphins in Port Phillip Bay. With a preference for schooling fish such as anchovies, the common dolphins' general cooperative foraging behaviour, in conjunction with familiarity with their habitat, the community may exploit patchy resources successfully thus facilitate their residency in the bay. However, prey targeted by common dolphins is likely not to be abundant enough to sustain a larger dolphin community in the bay, and resource competition with little penguins [87] and resource overlap with bottlenose dolphins [90] may contribute to this. It is possible that prey requirements of the female-dominated adult community, along with the requirements of calves and sub-adult dolphins, may represent the current carrying capacity for common dolphins in this urbanized bay.

Photo-identification images revealed that the dorsal fins of common dolphins in Port Phillip Bay were distinct enough to reliably identify every adult individual in the community. No unmarked adult common dolphins were found in the community. Of the 13 adult dolphins, 11 had considerable markings along the trailing edge of their dorsal fin and two showed few markings on their dorsal fins but were distinct in their coloration pattern. Furthermore, the dorsal fin coloration pattern of adult dolphins remained stable over time. A total of 14 calves were born in the common dolphin community during the study period, and calves could only be identified while still dependent on their mothers, based on the mother's dorsal fin markings. Calves and sub-adults in the bay showed generally no markings on their dorsal fins and were non-distinct in coloration pattern, and thus were not included in the analysis.

This study supports the findings of Neumann *et al.* [57] and Bearzi *et al.* [91,92] that adult common dolphins can be individually identified using dorsal fin images, similar to bottlenose dolphins. Evidence from this study suggests that photo-identification can also be used to reliable identify adults in larger common dolphin communities or populations. The ability to identify individual common dolphins in Port Phillip Bay is central to clarifying residency of this species to the bay and for an on-going monitoring of the resident dolphin community.

Ten of the adults identified as part of the Port Phillip Bay common dolphin community were females (repeatedly accompanied by calves and/or mammary slits present) and one a male (photographed postanal hump). The gender of two of the adult common dolphins could not be determined. The female-dominated Port Phillip Bay community differs from the gender composition of schools of common dolphins in the population found in shelf, coastal and gulf waters outside of Port Phillip Bay. There, a sociogenetic analyses of 62 schools of common dolphins revealed no significant difference from a 1 : 1 sex ratio in schools [93]. By contrast, genetic analysis of short-beaked common dolphins at a single stranding event in the English Channel in northern Europe revealed sex segregation for this species. A total of 52 female dolphins stranded, and the only male in the group was a calf [94]. Thus, drivers for gender composition of common dolphin schools remain unclear and may be related to the habitat they occur in and availability of prey. Similar to bottlenose dolphins (*Tursiops* spp.) that inhabit inshore habitat and bays around the world (e.g. Port Stephens and Jervis Bay in eastern Australia [95], and Sarasota Bay in Florida, USA (e.g. [56]), female common dolphins in Port Phillip Bay may benefit more from resource familiarity than males [96], potentially explaining the here observed female-biased sex ratio. The long-term and probable year-round residency of adult common dolphins in Port Phillip Bay and larger number of females than males suggest that the community may exhibit female philopatry. Resource familiarity probably increases female foraging success and as a result tends to increase reproductive fitness and success in rearing young [95,97].

Female philopatry occurs when males disperse while females stay in the area where they were born [98]. Delphinids show different levels of sex-biased dispersal around the world depending on species, and dispersal patterns may even differ between populations of the same species (e.g. [99,100]). Common dolphins that inhabit offshore waters tend to display no sex bias in dispersal, i.e. male and female common dolphins disperse similarly (e.g. [41,96,101,102]). Conversely, the high number of females and low number of males in the resident common dolphin community of Port Phillip Bay may be a result of sex-biased dispersal, where males may leave the bay and females remain resident. Thus, the potential female philopatry of the common dolphin community in Port Phillip Bay resembles the dispersal patterns of inshore bottlenose dolphin communities (e.g. [26,95,100]) more than that of other common dolphin communities.

The level of genetic exchange of the common dolphin community inside Port Phillip Bay with the previously identified larger Management Unit of common dolphins outside the bay (MU4 in [43]) is currently unknown. Common dolphins from Port Phillip Bay were not included in Bilgmann *et al.* [43], a study that assessed the genetic connectivity of this species in waters off southern and south-eastern Australia. However, because of the small size of the local common dolphin community in Port Phillip Bay, it is expected that some genetic exchange exists with the population of common dolphins outside the bay, potentially mediated via male-biased dispersal (i.e. males visiting the bay to interbreed with local females). This potentially facilitates sufficient genetic exchange to avoid inbreeding and allow long-term sustainability of the common dolphin community in the bay.

Resources in inshore waters are likely to be more predictable than in offshore or pelagic waters [103]. In mammals, predictability of food resources is particularly important for females due to their increased energy requirements [98]. Captive female bottlenose dolphins increase their food intake when lactating by 52% for *Tursiops aduncus* [104] and by 58–97% for *Tursiops truncatus* [105]. Free-ranging common dolphins in Port Phillip Bay may also increase their food intake when lactating, and may benefit from resource familiarity. Besides the predictability of prey, the fat content of available prey may also play an important role for common dolphins. For example, common dolphins in the Bay of Biscay in the north-eastern Atlantic Ocean select fish that have a high fat content to meet the needs of their highly energetic behaviour. Fish that have a high fat content in the Bay of Biscay include sardines (*Sardina pilchardus*), anchovies (*Engraulis encrasicolus*), sprat (*Sprattus sprattus*) and horse mackerel (*Trachurus* spp), and provide up to 89% of the energy requirements of the common dolphins there [106]. Closely related fish species of the same family to those found in the Bay of Biscay are found in Port Phillip Bay, including sardines (*Sardinops sagax*), anchovies (*Eugraulis australis*), sandy sprat (*Hyperlophus vittatus*) and jack mackerel (*Trachurus declivis*) (e.g. [107,108]). Anchovies are one of the preferred prey species of common dolphins in South Australian waters [83] and are likely to be the main target species for Port Phillip Bay common dolphins, potentially because of their high abundance. Therefore, the energy requirements of the common dolphin community in Port Phillip Bay may be met by the predictability of prey through resource familiarity and by consuming fish species with higher fat content.

Generally, common dolphins are considered an offshore species [109,110] that can also be found in waters over the continental shelf [40–43]. The species has been documented to occur in deep continental shelf waters in the Alboran Sea in southern Spain, ranging from 25 to 1300 m [40], and in waters over ‘the Gully’, a submarine canyon in Nova Scotia, Canada, ranging from 1000 to 2500 m [111]. In some areas around the world, this species has been found closer to shore and in shallower waters. For example, in the Hauraki Gulf, New Zealand, common dolphins were found in water depths between 7 and 52 m [112], in the Gulf St Vincent, South Australia between 14 and 40 m, [52], in the Gulf of California, Mexico, between 3 and 105 m [113] and in the Moray Firth, Scotland, between 51 and 209 m [114]. Common dolphins in Port Phillip Bay were regularly encountered along the eastern coast in waters close to shore in depths of 4 to 21 m. During systematic surveys, 50% of the common dolphin sightings were within 2.2 km of the coast. The range of water depths in which the Port Phillip common dolphins were encountered was more restricted than those reported for this species elsewhere in the world, probably because of the distinct underwater topography only found in the south-eastern region of the bay. A preference for shallow water depths and close proximity to the coast is atypical for this species of common dolphins, and rather typical for other inshore delphinids such as Hector's dolphins (e.g. [115,116]), bottlenose dolphins (*Tursiops* spp*.*) (e.g. [26,53,95]) and humpback dolphins (*Sousa chinensis*) (e.g. [117,118]). As a result, the common dolphins in Port Phillip Bay may be exposed to the same threats that other inshore dolphin communities are exposed to close to heavily urbanized coasts.

Common dolphins in Port Phillip Bay were observed less often during the warmer months, but it is unclear whether this was due to a reduced survey effort, a shift of habitat use within the bay or due to the individuals temporarily leaving the bay. Changes in near-shore distribution may be a result of seasonal prey movement (e.g. [119]). Local anchovy schools are thought to move inshore and form denser schools during the cooler months (Phil McAdam, Vancouver Fisheries, Port Phillip Bay 2015, personal communication), which potentially influenced common dolphin distribution in a way that led to more re-sightings during periods of cooler water temperature.

Challenges in the analysis of the study presented here included the variation in survey design over the study period and inconsistencies in the conduction of surveys across all months of the year. This led to several limitations in the data. The data from the different surveys (systematic and non-systematic) were not directly comparable; only 21 of the 48 surveys met the assumptions of conventional distance sampling [65]. Non-systematic surveys, consisting of haphazard and random-line survey routes, did not cover the survey area as extensively as the systematic surveys and did not allow for the equal coverage probability of points within the area. Furthermore, the offshore systematic surveys extended out to approximately 10 km from the coast, with the furthest observation made at 9.3 km. By contrast, the furthest distance from the coast that the common dolphins were observed during non-systematic survey was 3.8 km. This is probably a result of the lesser area covered by the non-systematic surveys when compared with the inshore and offshore systematic surveys. Despite the limitations resulting from variable survey design, this study gave sufficient evidence for the conclusions presented here. However, we recommend that future research uses systematic line-transect surveys and that, at a minimum, all survey effort is recorded (speed, transect routes, and time spent on and off survey). This would allow for a collection of additional observational data in all water depths to enable quantification of habitat use and seasonal movement of the resident common dolphin community in Port Phillip Bay.

### 5.1. Management implications

A number of human activities have the potential to impact common dolphins in urbanized Port Phillip Bay. Threats that have been identified for the resident southern Australian bottlenose dolphin community include recreational and commercial fishing, commercial shipping and industrial activity [30]; these activities are also likely to impact the resident common dolphin community in the bay. Commercial fishing and purse-seine netting in Port Phillip Bay is currently strictly regulated under the *Fisheries Act 1995* [120]. As of 1 April 2016, commercial fishing has been phased out in Port Phillip Bay [121], reducing the risk of common dolphin prey depletion and entanglement. Other potential threats to the common dolphins in Port Phillip Bay include boat strikes [9], disruptions to feeding, resting and socializing behaviours due to vessel interaction (e.g. [122–126]), bioaccumulation of toxins such as mercury [15] and the entanglement and ingestion of recreational fishing debris [5]. Although the minimum approach distance of 100 m of vessels to dolphins in Victorian waters is legislated and enforced under the *Victorian Wildlife (Marine Mammal) Regulations 2009* [127], boat strikes of common dolphins, in particular common dolphin calves, can and have occurred in Port Phillip Bay. Furthermore, interactions with recreational fishing gear that can lead to serious injury and/or mortality [5] are also of concern. Accordingly, management of the inshore, common dolphin community residing in the shallow urbanized Port Phillip Bay should be considered separately to other common dolphin communities.

The residency of around 30 common dolphins (including adults, sub-adults and calves) to the relatively shallow and urbanized Port Phillip Bay is atypical for this species. The proximity to humans in the bay makes this small dolphin community particularly vulnerable to anthropogenic impacts. A further concern is the sustainability of such a small number of dolphins in the embayment given that the level of genetic exchange with dolphins outside the bay is unknown. Future research is needed to clarify the level of gene flow of the resident common dolphin community with common dolphins outside the bay, and the genetic diversity within the community. This is important because if gene flow is severely reduced for the small resident Port Phillip Bay common dolphin community, inbreeding may occur potentially reducing the dolphins' reproductive fitness. Low genetic diversity may also reduce the ability of the resident common dolphin community to adapt to human-induced impacts and/or environmental change thus reducing chances of long-term sustainability in the bay.

This study provides evidence of residency of a small common dolphin nursery community in Port Phillip Bay, south-eastern Australia. The semi-enclosed nature of the bay, the common dolphins' shallow water habitat preferences and close proximity to an urbanized coast potentially expose them to additional threats not faced by typical offshore common dolphin communities. The threats common dolphins are exposed to in Port Phillip Bay are similar to those of resident coastal bottlenose dolphins in the bay.

It is proposed that due to the low number of individuals in the resident Port Phillip Bay common dolphin community, the unique habitat occupancy and high proportion of breeding females, the community should be considered and managed separately to the common dolphin management units in coastal and shelf waters outside of the bay. Management approaches should aim at facilitating the common dolphins' long-term residency to Port Phillip Bay by managing human-induced impacts in the bay, maximizing genetic exchange with dolphins outside of the bay, and by on-going monitoring of the resident common dolphin community.

## Data accessibility

The dataset supporting this article is available on the Dryad Digital Repository at http://dx.doi.org/10.5061/dryad.5j1k1 [128].

## References

[1] Gowans S, Würsig B, Karczmarski L 2007 The social structure and strategies of delphinids: predictions based on an ecological framework. Adv. Mar. Biol. 53, 195–294. (doi:10.1016/S0065-2881(07)53003-8) 17936137 10.1016/S0065-2881(07)53003-8

[2] Whitehead H, Mann J 2000 Female reproductive strategies of cetaceans. Cetacean societies: field studies of dolphins and whales, pp. 219–246. Chicago, IL: University of Chicago Press.

[3] Bearzi G, Agazzi S, Gonzalvo J, Costa M, Bonizzoni S, Politi E, Piroddi C, Reeves R 2008 Overfishing and the disappearance of short-beaked common dolphins from western Greece. Endangered Species Res. 5, 1–12. (doi:10.3354/esr00103)

[4] Piroddi C, Bearzi G, Gonzalvo J, Christensen V 2011 From common to rare: the case of the Mediterranean common dolphin. Biol. Conserv. 144, 2490–2498. (doi:10.1016/j.biocon.2011.07.003)

[5] Wells RS, Hofmann S, Moors TL 1998 Entanglement and mortality of bottlenose dolphins, *Tursiops truncatus*, in recreational fishing gear in Florida. Fish. Bull. 96, 647–650.

[6] Kemper CM, Flaherty A, Gibbs SE, Hill M, Long M, Byard R 2005 Cetacean captures, strandings and mortalities in South Australia 1881–2000, with special reference to human interactions. Aust. Mamm. 27, 37–47. (doi:10.1071/AM05037)

[7] Nowacek SM, Wells RS, Solow AR 2001 Short-term effects of boat traffic on bottlenose dolphins *Tursiops truncatus*, in Sarasota Bay, Florida. Mar. Mamm. Sci. 17, 673–688. (doi:10.1111/j.1748-7692.2001.tb01292.x)

[8] Bejder L, Samuels A, Whitehead H, Gales N 2006 Interpreting short-term behavioural responses to disturbance within a longitudinal perspective. Anim. Behav. 72, 1149–1158. (doi:10.1016/j.anbehav.2006.04.003)

[9] Martinez E, Stockin KA 2013 Blunt trauma observed in a common dolphin *Delphinus* sp. likely caused by a vessel collision in the Hauraki Gulf, New Zealand. Pacific Conserv. Biol. 19, 19–27. (doi:10.1071/PC130019)

[10] Jensen FH, Bejder L, Wahlberg M, Aguilar Soto N, Johnston DW, Madsen PT 2009 Vessel noise effects on delphinid communication. Mar. Ecol. Progr. Ser. 395, 161–175. (doi:10.3354/meps08204)

[11] Buckstaff KC 2004 Effects of watercraft noise on the acoustic behaviour of bottlenose dolphins *Tursiops truncatus*, in Sarasota Bay, Florida. Mar. Mamm. Sci. 20, 709–725. (doi:10.1111/j.1748-7692.2004.tb01189.x)

[12] Bearzi G *Delphinus delphis* (Mediterranean subpopulation). IUCN Red List of Threatened Species 2003. See www.iucnredlist.org (accessed 5 June 2016).

[13] Kucklick J et al. 2011 Bottlenose dolphins as indicators of persistent organic pollutants in the western North Atlantic Ocean and northern Gulf of Mexico. Environ. Sci. Technol. 45, 4270–4277. (doi:10.1021/es1042244) 21526819 10.1021/es1042244

[14] Balmer BC et al. 2011 Relationship between persistent organic pollutants (POPs) and ranging patterns in common bottlenose dolphins (*Tursiops truncatus*) from coastal Georgia, USA. Sci. Total Environ. 409, 2094–2101. (doi:10.1016/j.scitotenv.2011.01.052) 21356543 10.1016/j.scitotenv.2011.01.052

[15] Monk A, Charlton-Robb K, Buddhadasa S, Thompson RM 2014 Comparison of mercury contamination in live and dead dolphins from a newly described species, *Tursiops australis*. PLoS ONE 9, e0104887 (doi:10.1371/journal.pone.0104887) 10.1371/journal.pone.0104887 PMC4138083 25137255

[16] Wilson B, Grellier K, Hammond PS, Brown G, Thompson PM 2000 Changing occurrence of epidermal lesions in wild bottlenose dolphins. Mar. Ecol. Progr. Ser. 205, 283–290. (doi:10.3354/meps205283)

[17] Wilson B, Thompson PM, Hammond PS 1997 Skin lesions and physical deformities in bottlenose dolphins in the Moray Firth: Population and prevalence and age-sex differences. Ambio 26, 243.

[18] Ford JKB, Ellis GM, Balcomb KC 2000 Killer whales, second edition. Vancouver, Canada: UBC Press.

[19] Bejder L, Dawson S 2001 Abundance, residency, and habitat utilisation of Hector's dolphins (*Cephalorhynchus hectori*) in Porpoise Bay, New Zealand. N. Z. J. Mar. Freshw. Res. 35, 277–287. (doi:10.1080/00288330.2001.9516998)

[20] Parsons E 1998 The behaviour of Hong Kong's resident cetaceans: the Indo-Pacific hump-backed dolphin and the finless porpoise. Aquat. Mamm. 24, 91–110.

[21] Elliser CR, Herzing DL 2014 Long-term social structure of a resident community of Atlantic spotted dolphins, *Stenella Frontalis*, in the Bahamas 1991–2002. Mar. Mamm. Sci. 30, 308–328. (doi:10.1111/mms.12039)

[22] Scott MD, Irvine BA, Wells RS 1990 A long-term study of bottlenose dolphins on the west coast of Florida. In The bottlenose dolphin (eds Leatherwood S, Reeves R), pp.~235–244. San Diego, CA: Academic Press.

[23] Berrow SD, Holmes B, Kiely OR 1996 Distribution and abundance of bottle-nosed dolphins *Tursiops truncatus* (Montagu) in the Shannon Estuary. Biol. Environ. Proc R. Irish Acad. B 96, 1–9.

[24] Hammond P, Thompson P 1991 Minimum estimate of the number of bottlenose dolphins *Tursiops truncatus* in the Moray Firth, NE Scotland. Biol. Conserv. 56, 79–87. (doi:10.1016/0006-3207(91)90090-V)

[25] Smolker RA, Richards AF, Connor RC, Pepper JW 1992 Sex differences in patterns of association among Indian Ocean bottlenose dolphins. Behav. 123, 38–69. (doi:10.1163/156853992X00101)

[26] Krützen M, Sherwin WB, Berggren P, Gales N 2004 Population structure in an inshore cetacean revealed by microsatellite and mtDNA analysis: bottlenose dolphins (*Tursiops* sp.) in Shark Bay, Western Australia. Mar. Mamm. Sci. 20, 28–47. (doi:10.1111/j.1748-7692.2004.tb01139.x)

[27] Möller LM, Allen SJ, Harcourt RG 2002 Group characteristics, site fidelity and abundance of bottlenose dolphins (*Tursiops aduncus*) in Jervis Bay and Port Stephens, southeastern Australia. Aust. Mamm. 24, 11–21. (doi:10.1071/AM02011)

[28] Chabanne D, Finn H, Salgado-Kent C, Bejder L 2012 Identification of a resident community of bottlenose dolphins (*Tursiops aduncus*) in the Swan Canning Riverpark, Western Australia, using behavioural information. Pacific Conser. Biol. 18, 247–262. (doi:10.1071/PC120247)

[29] Fury CA, Harrison PL 2008 Abundance, site fidelity and range patterns of Indo-Pacific bottlenose dolphins (*Tursiops aduncus*) in two Australian subtropical estuaries. Mar. Freshw. Res. 59, 1015–1027. (doi:10.1071/MF08109)

[30] Charlton K, Taylor AC, McKechnie SW 2007 A note on divergent mtDNA lineages of bottlenose dolphins from coastal waters of southern Australia. J. Cetacean Res. Manage. 8, 173.

[31] Warren-Smith ÁB, Dunn WL 2006 Epimeletic behaviour toward a seriously injured juvenile bottlenose dolphin (*Tursiops* sp.) in Port Phillip, Victoria, Australia. Aquat. Mamm. 32, 357 (doi:10.1578/AM.32.3.2006.357)

[32] Zanardo N, Parra GJ, Möller LM 2015 Group characteristics, abundance and site fidelity of Burrunan dolphins (‘*Tursiops australis*’) in Adelaide's metropolitan waters. South Aust. Nat. 89, 55–60.

[33] Zanardo N, Parra GJ, Möller LM In press. Site fidelity, residency, and abundance of bottlenose dolphins (Tursiops sp.) in Adelaide's coastal waters, South Australia. Mar. Mamm. Sci. (doi:10.1111/mms.12335)

[34] Charlton-Robb K, Gershwin L-A, Thompson R, Austin J, Owen K, McKechnie S 2011 A new dolphin species, the Burrunan dolphin *Tursiops australis* sp. nov., endemic to southern Australian coastal waters. PLoS ONE 6, e24047 (doi:10.1371/journal.pone.0024047) 21935372 10.1371/journal.pone.0024047 PMC3173360

[35] Möller LM, Bilgmann K, Charlton-Robb K, Beheregaray L 2008 Multi-gene evidence for a new bottlenose dolphin species in southern Australia. Mol. Phylogenet. Evol. 49, 674–681. (doi:10.1016/j.ympev.2008.08.011) 18789390 10.1016/j.ympev.2008.08.011

[36] Moura AE, Nielsen SC, Vilstrup JT, Moreno-Mayar JV, Gilbert MTP, Gray HW, Natoli A, Möller L, Hoelzel AR 2013 Recent diversification of a marine genus (*Tursiops* spp.) tracks habitat preference and environmental change. Syst. Biol. 62, 865–877. (doi:10.1093/sysbio/syt051) 23929779 10.1093/sysbio/syt051

[37] Committee on Taxonomy. 2016 List of marine mammal species and subspecies. Society for Marine Mammalogy. 2016 Last updated May 2016. See https://www.marinemammalscience.org/species-information/list-of-marine-mammal-species-subspecies/ (accessed 25 May 2016).

[38] Jefferson TA, Webber MA, Pitman RL 2015 Marine mammals of the World: a comprehensive guide to their identification, 2nd edn San Diego, CA: Academic Press.

[39] Jefferson TA, Webber MA, Pitman RL 2011 Marine mammals of the World: a comprehensive guide to their identification. San Diego, CA: Academic Press.

[40] Canadas A, Sagrminaga R, Garcica-Tiscar S 2002 Cetacean distribution related with depth and slope in the Mediterranean waters off Southern Spain. Deep-Sea Research. Part I Oceanogr. Res. Papers 11, 2053–2073. (doi:10.1016/S0967-0637(02)00123-1)

[41] Möller LM, Valdez PF, Allen S, Bilgmann K, Corrigan S, Beheregaray LB 2011 Fine-scale genetic structure in short-beaked common dolphins (*Delphinus delphis*) along the East Australian Current. Mar. Biol. 158, 113–126. (doi:10.1007/s00227-010-1546-x)

[42] Bilgmann K, Möller LM, Harcourt RG, Gales R, Beheregaray LB 2008 Common dolphins subject to fisheries impacts in Southern Australia are genetically differentiated: implications for conservation. Anim. Conserv. 11, 518–528. (doi:10.1111/j.1469-1795.2008.00213.x)

[43] Bilgmann K, Parra GJ, Zanardo N, Beheregaray LB, Möller LM 2014 Multiple management units of short-beaked common dolphins subject to fisheries bycatch off southern and southeastern Australia. Mar. Ecol. Progr. Press 500, 265–279. (doi:10.3354/meps10649)

[44] Hui CA 1979 Undersea topography and distribution of the genus *Delphinus* in the Southern Californa Bight. J. Mamm. 60, 521–527. (doi:10.2307/1380092)

[45] Oviedo L, Esteves MA, Acevedo R, Silva N, Bolaños-Jiménez J, Quevedo AM, Fernández M 2010 Abundance, distribution and behaviour of common dolphins, *Delphinus* spp., off north-eastern Venezuela: implications for conservation and management. J. Mar. Biol. Assoc. UK 90, 1623–1631. (doi:10.1017/S002531540999097X)

[46] Cockcroft VG, Peddemors VM 1990 Seasonal distribution and density of common dolphins *Delphinus delphis* off the south-east coast of southern Africa. S. Afr. J. Mar. Sci. 9, 371–377. (doi:10.2989/025776190784378853)

[47] Heyning JE, Perrin WF 1994 Evidence for two species of common dolphin (genus *Delphinus*) from the Eastern North Pacific. Contrib. Sci. 442, 1–35.

[48] Genov T, Bearzi G, Bonizzoni S, Tempesta M 2012 Long-distance movement of a lone short-beaked common dolphin *Delphinus delphis* in the central Mediterranean Sea. Mar. Biodivers. Rec. 5, e9 (doi:10.1017/S1755267211001163)

[49] White C 1999 Molecular systematics of the common dolphin, Delphinus delphis. Adelaide, Australia: University of Adelaide.

[50] Moura A, Natoli A, Rogan E, Hoelzel A 2013 Atypical panmixia in a European dolphin species (*Delphinus delphis*): implications for the evolution of diversity across oceanic boundaries. J. Evol. Biol. 26, 63–75. (doi:10.1111/jeb.12032) 23205921 10.1111/jeb.12032

[51] Kemper CM, Gibbs SE 2001 Dolphin interactions with tuna feedlots at Port Lincoln, South Australia and recommendations for minimising entanglements. J. Cetacean Res. Manage. 3, 283–292.

[52] Filby NE, Bossely M, Sanderson KJ, Martinez E, Stockin KA 2010 Distribution and population demographics of common dolphins (*Delphinus delphis*) in the Gulf St. Vincent, South Australia. Aquat. Mamm. 36, 33–45. (doi:10.1578/AM.36.1.2010.33)

[53] Gubbins C 2002 Use of home range by resident bottlenose dolphins (*Tursiops truncatus*) in a South Carolina estuary. J. Mamm. 83, 178–187. (doi:10.1644/1545-1542(2002)083<0178:UOHRBR>2.0.CO;2)

[54] Wilson B, Thompson P, Hammond P 1997 Habitat use by bottlenose dolphins: seasonal distribution and stratified movement patterns in the Moray Firth, Scotland. J. Appl. Ecol. 34, 1365–1374. (doi:10.2307/2405254)

[55] Defran R, Shultz GM, Weller DW 1990 A technique for the photographic identification and cataloging of dorsal fins of the bottlenose dolphin (*Tursiops truncatus*). Individual Recognition of Cetaceans: Use of Photo-Identification and Other Techniques to Estimate Population Parameters. In *Individual recognition of cetaceans: use of photo-identification and other techniques to estimate population parameters*. Report of the International Whaling Commission, special issue 12 (eds PS Hammond, SA Mizroch, GP Donovan), pp. 53–55. Cambridge, UK: IWC.

[56] Wells RS, Scott MD 1990 Estimating bottlenose dolphin population parameters from individual identification and capture-release techniques. Individual recognition of cetaceans: use of photo-identification and other techniques to estimate population parameters. *Reports of the International Whaling Commission*, special issue 12, pp. 407–415. Cambridge, UK: IWC.

[57] Neumann DR, Leitenberger A, Orams MB 2002 Photo-identification of short-beaked common dolphins (*Delphinus delphis*) in north-east New Zealand: a photo-catalogue of recognisable individuals. N. Z. J. Mar. Freshw. Res. 36, 593–604. (doi:10.1080/00288330.2002.9517115)

[58] C.S.I.R.O. 1996 Port Phillip Bay Environmental Study – Final Report Dickson, ACT: CSIRO Australia.

[59] Australian Bureau of Statistics. 2015 See http://www.abs.gov.au (accessed 1 April 2015).

[60] Holdgate G, Geurin B, Wallace M, Gallagher S 2001 Marine geology of Port Phillip, Victoria. Aust. J. Earth Sci. 48, 439–455. (doi:10.1046/j.1440-0952.2001.00871.x)

[61] Bird ECF 2011 Changes on the coastline of Port Phillip Bay. Melbourne, Australia: Office of the Environmental Monitor.

[62] Morris L, Ball D 2006 Habitat suitability modelling of economically important fish species with commercial fisheries data. ICES J. Mar. Sci. 63, 1590–1603. (doi:10.1016/j.icesjms.2006.06.008)

[63] Bird ECF 2010 Victoria: Port Phillip Bay (Point Lonsdale to Point Nepean). In Encyclopedia of the World's coastal landforms (ed. Bird ECF), pp. 1337–1348. Berlin, Germany: Springer.

[64] C-Map. 2006 PC-Planner. 11.02 ed. Italy.

[65] Buckland ST, Anderson DR, Burnham KP, Laake JL, Borchers DL, Thomas L 2009 Introduction to distance sampling: estimating abundance of biological populations. Oxford, UK: Oxford University Press.

[66] Thomas L et al. 2009 Distance 6.0. Release 2. Research Unit for Wildlife Population Assessment. St Andrews, UK: University of St. Andrews.

[67] Dawson S, Paul W, Slooten E, Barlow J 2008 Design and field methods for sighting surveys of cetaceans in coastal and riverine habitats: Publications, Agencies and Staff of the U.S. Department of Commerce.

[68] Shane SH 1990 Behaviour and ecology of the bottlenose dolphin at Sanibel Island, Florida. In The bottlenose dolphin (eds Leatherwood S, Reeves R). San Diego, CA: Academic Press.

[69] Würsig B, Würsig M 1977 The photographic determination of group size, composition and stability of coastal porpoises (*Tursiops truncatus*). Science 198, 755–756. (doi:10.1126/science.198.4318.755)

[70] Würsig B, Jefferson TA 1990 Methods of photo-identification for small cetaceans. In *Individual recognition of cetaceans: use of photo-identification and other techniques to estimate population parameters*. Report of the International Whaling Commission, special issue 12 (eds PS Hammond, SA Mizroch, GP Donovan), pp. 43–52. Cambridge, UK: IWC.

[71] Neumann DR, Russell K, Orams MB, Baker SC, Duignan P 2002 Identifying sexually mature, male short-beaked common dolphins (*Delphinus delphis*) at sea, based on the presence of a postanal hump. Aquat. Mamm. 28, 181–187.

[72] Murphy S, Collet A, Rogan E 2005 Mating strategy in the male common dolphin (*Delphinus delphis*): what gonadal analysis tells us. J. Mamm. 86, 1247–1258. (doi:10.1644/1545-1542(2005)86[1247:MSITMC]2.0.CO;2)

[73] Rosel PE et al. 2011 Photoidentification capture-mark-recapture techniques for estimating abundance of bay, sound and estuary populations of bottlenose dolphins along the US East Coast and Gulf of Mexico: a workshop report. NOAA Technical Memorandum NMFS-SEFSC 621, 30.

[74] Urian KW, Hohn AA, Hansen LJ 1999 Status of the photo-identification catalog of coastal bottlenose dolphins of the western North Atlantic: report of a workshop of catalog contributors. Beaufort, NC: US Department of Commerce, National Oceanic and Atmospheric Administration, National Marine Fisheries Service, Southeast Fisheries Science Center, NOAA Beaufort Laboratory.

[75] Urian KW et al. 2015 Recommendations for photo-identification methods used in capture-recapture models with cetaceans. Mar. Mamm. Sci. 31, 298–321. (doi:10.1111/mms.12141)

[76] Kiszka J, Simon-Bouhet B, Gastebois C, Pusineri C, Ridoux V 2012 Habitat partitioning and fine scale population structure among insular bottlenose dolphins (*Tursiops aduncus*) in a tropical lagoon. J. Exp. Mar. Biol. Ecol. 416, 176–184. (doi:10.1016/j.jembe.2012.03.001)

[77] E.S.R.I. 2013 ArcGIS Desktop: Release 10.2. Redlands, CA: Environmental Systems Research Institute.

[78] Service AH. 2012 Australian ENC—Port Phillip and Western Port. Australian Hydrographic Service, Commonwealth of Australia, Licence no 3490SL.

[79] R Development Core Team. 2015 R: a language and environment for statistical computing. 3.2.0 ed. Vienna, Austria: R Foundation for Statistical Computing.

[80] Scarparci C, Bigger SW, Saville TA, Nugegoda D 1999 A rare sighting of the common dolphin *Delphinus delphis* in Port Phillip, Victoria. The Victorian Nat. 116, 65–67.

[81] Warneke RM 1996 Common dolphin. In Mammals of Victoria: distribution, ecology and conservation. (ed. Menkhurst P). South Melbourne, Australia: Oxford University Press.

[82] Pusineri C, Magnin V 2007 Food and feeding ecology of the common dolphin (*Delphinus delphis*) in the oceanic northeast Atlantic and comparison with its diet in the neritic areas. Mar. Mamm. Sci. 23, 30–47. (doi:10.1111/j.1748-7692.2006.00088.x)

[83] Gibbs SE 2007 Multi-species niche partioning on high trophic level marine predators in South Australia. Sydney, Australia: Macquarie University.

[84] Meynier L, Stockin KA, Bando M, Duignan PJ 2008 Stomach contents of common dolphin (*Delphinus* sp.) from New Zealand waters. N. Z. J. Mar. Freshw. Res. 42, 257–268. (doi:10.1080/00288330809509952)

[85] Neumann DR, Orams MB 2003 Feeding behaviours of short-beaked common dolphins, *Delphinus delphis*, in New Zealand. Aquat. Mamm. 29.1, 137–149. (doi:10.1578/016754203101023997)

[86] Jenkins GP, McKinnon L 2006 Channel Deepening Supplemenatry Environmental Effects Statement—Aquaculture and Fisheries: Primary Industries Research, Victoria, Queenscliff.

[87] Chiaradia A, Forero MG, Hobson KA, Swearer SE, Hume F, Renwick L, Dann P 2011 Diet segregation between two colonies of little penguins *Eudyptula minor* in southeast Australia. Aust. Ecol. 37, 610–619. (doi:10.1111/j.1442-9993.2011.02323.x)

[88] McCutcheon C, Dann P, Salton M, Renwick L, Hoskins AJ, Gormley AM, Arnould JPY 2011 The foraging range of Little Penguins (*Eudyptula minor*) during winter. Emu 111, 321–329. (doi:10.1071/MU10078)

[89] Gormley AM, Dann P 2009 Examination of little penguin winter movements from satellite tracking data. Victoria, Australia: Department of Sustainability and Environment.

[90] Gibbs SE, Harcourt RG, Kemper CM 2011 Niche differentiation of bottlenose dolphin species in South Australia revealed by stable isotopes and stomach contents. Wildl. Res. 38, 261–270. (doi:10.1071/WR10108)

[91] Bearzi G, Politi E, Agazzi S, Bruno S, Costa M, Bonizzoni S 2005 Occurrence and presence status of coastal dolphins (*Delphinus delphis and Tursiops truncatus*) in the eastern Ionian Sea. Aquat. Conserv. Mar. Freshw. Ecosyst. 15, 243–257. (doi:10.1002/aqc.667)

[92] Bearzi G, Bonizzoni S, Agazzi S, Gonzalvo J, Currey R 2011 Striped dolphins and short-beaked common dolphins in the Gulf of Corinth, Greece: abundance estimates from dorsal fin photographs. Mar. Mamm. Sci. 27, E165–E184. (doi:10.1111/j.1748-7692.2010.00448.x)

[93] Zanardo N, Bilgmann K, Parra GJ, Möller LM 2016 Socio-genetic structure of short-beaked common dolphins in southern Australia. J. Zool. 299, 89–97. (doi:10.1111/jzo.12330)

[94] Viricel A, Strand AE, Rosel PE, Garcia P 2008 Insights on common dolphin (*Delphinus delphis*) social organization from genetics analysis of a mass-stranded pod. Behav. Ecol. Sociobiol. 63, 173 (doi:10.1007/s00265-008-0648-7)

[95] Möller LM, Beheregaray LB 2004 Genetic evidence for sex-biased dispersal in resident bottlenose dolphins (*Tursiops aduncus*). Mol. Ecol. 13, 1607–1612. (doi:10.1111/j.1365-294X.2004.02137.x) 15140103 10.1111/j.1365-294X.2004.02137.x

[96] Möller LM 2011 Sociogenetic structure, kin associations and bonding in delphinids. Mol. Ecol. 21, 745–764. (doi:10.1111/j.1365-294X.2011.05405.x) 22212106 10.1111/j.1365-294X.2011.05405.x

[97] Connor R, Wells R, Mann J, Read A 2000 The bottlenose dolphin: social relationships in a fission-fusion society. In Cetacean societies: field studies of whales and dolphins (eds J Mann, RC Connor, PL Tyack PL, H Whitehead), pp. 91–125. Chicago, IL: The University of Chicago Press.

[98] Clutton-Brock T, Lukas D 2012 The evolution of social philopatry and dispersal in female mammals. Mol. Ecol. 21, 472–492. (doi:10.1111/j.1365-294X.2011.05232.x) 21883582 10.1111/j.1365-294X.2011.05232.x

[99] Natoli A, Birkun A, Aguilar A, Lopez A, Hoelzel AR 2005 Habitat structure and the dispersal of male and female bottlenose dolphins (*Tursiops truncatus*). Proc. R. Soc. B 272, 1217–1226. (doi:10.1098/rspb.2005.3076) 10.1098/rspb.2005.3076 PMC1564106 16024385

[100] Wiszniewski J, Beheregaray LB, Allen SJ, Möller LM 2010 Environmental and social influences on the genetic structure of bottlenose dolphins (*Tursiops aduncus*) in Southeastern Australia. Conserv. Genet. 11, 1405–1419. (doi:10.1007/s10592-009-9968-z)

[101] Bilgmann K, Möller L, Harcourt R, Gales R, Beheregaray L 2009 Reply to ‘Clarifying the interpretation of Hamer *et al*. (2008) by Bilgmann *et al*. (2008)’. Anim. Conserv. 12, 289–290. (doi:10.1111/j.1469-1795.2009.00270.x)

[102] Natoli A, Canadas A, Peddemors VM, Aguliar A, Vaquero C, Fernandez-Piqueras P, Hoelzel AR 2006 Phylogeography and alpha taxonomy of the common dolphin (*Delphinus sp*.). J. Evol. Biol. 19, 943–954. (doi:10.1111/j.1420-9101.2005.01033.x) 16674590 10.1111/j.1420-9101.2005.01033.x

[103] Möller L, Parra GJ, Bilgmann K 2012 Population size, structure and habitat preferences of common dolpins in South Australia: enhancing the assessment, reduction and mitigation of fisheries operational interactions. Hobart, Australia: Australian Marine Mammal Centre, Australian Antarctic Division.

[104] Cheal AJ, Gales NJ 1991 Body mass and food intake in captive, breeding bottlenose dolphins, *Tursiops truncatus*. Zoo Biol. 10, 451–456. (doi:10.1002/zoo.1430100603)

[105] Kastelein RA, Vaughan N, Walton S, Wiepkema PR 2002 Food intake and body measurements of Atlantic bottlenose dolphins (*Tursiops truncatus*) in captivity. Mar. Environ. Res. 53, 199–218. (doi:10.1016/S0141-1136(01)00123-4) 11824828 10.1016/s0141-1136(01)00123-4

[106] Meynier L, Pusineri C, Spitz J, Santos MB, Pierce GJ, Ridoux V 2008 Intraspecific dietary variation in the short-beaked common dolphin *Delphinus delphis* in the Bay of Biscay: importance of fat fish. Mar. Ecol. Progr. Ser. 354, 277 (doi:10.3354/meps07246)

[107] Chiaradia A, Costalunga A, Kerry K 2003 The diet of little penguins (*Eudyptula minor*) at Phillip Island, Victoria, in the absence of a major prey–Pilchard (*Sardinops sagax*). Emu 103, 43–48. (doi:10.1071/MU02020)

[108] Bunce A 2001 Prey consumption of Australasian gannets (*Morus serrator*) breeding in Port Phillip Bay, southeast Australia, and potential overlap with commercial fisheries. ICES J. Mar. Sci. 58, 904–915. (doi:10.1006/jmsc.2001.1083)

[109] Perrin WF 2002 Common dolphins *Delphinus delphinus D. capensis* and *D. tropicalis*. In Encyclopedia of marine mammals (eds Perrin WF, Würsig B, Thewissen JGM), pp. 245–248. San Diego, CA: Academic Press.

[110] Perrin WF 2009 Common dolphins: *Delphinus delphis* and *D. capensis*. In Encyclopedia of marine mammals, 2nd edn (eds Perrin WF, Würsig B, Thewissen JGM), pp. 255–259. San Diego, CA: Academic Press.

[111] Distribution and habitat partitioning by small odontocetes in the Gully, a submarine canyon on the Scotian Shelf. Can. J. Zool. 73, 1599–1608. (doi:10.1139/z95-190)

[112] Stockin KA, Pierce GJ, Binedell V, Wisemen N, Orams MB 2008 Factors affecting the occurrence and demographics of common dolphins (*Delphinus* sp.) in the Hauraki Gulf, New Zealand. Aquat. Mamm. 2008, 2 (doi:10.1578/am.34.2.2008.200)

[113] Silber GK, Newcomer MW, Silber PC, Perez-Cortes H, Ellis GM 1994 Cetaceans of the northern Gulf of California: distribution, occurrence, and relative abundance. Mar. Mamm. Sci. 10, 283–298. (doi:10.1111/j.1748-7692.1994.tb00483.x)

[114] Robinson KP, Eisfeld SM, Costa M, Simmonds MP 2010 Short-beaked common dolphin (*Delphinus delphis*) occurrence in the Moray Firth, north-east Scotland. Mar. Biodivers. Rec. 3, e55 (doi:10.1017/S1755267210000448)

[115] Dawson SM 1991 Incidental catch of Hector's dolphin in inshore gillnets. Mar. Mamm. Sci. 7, 283–295. (doi:10.1111/j.1748-7692.1991.tb00103.x)

[116] Rayment W, Dawson S, Slooten E 2010 Seasonal changes in distribution of Hector's dolphin at Banks Peninsula, New Zealand: implications for protected area design. Aquat. Conserv. Mar. Freshw. Ecosyst. 20, 106–116.

[117] Karczmarski L, Cockcroft VG, McLachlan A 2000 Habitat use and preferences of Indo-Pacific humpback dolphins *Sousa chinensis* in Algoa Bay, South Africa. Mar. Mamm. Sci. 16, 65–79. (doi:10.1111/j.1748-7692.2000.tb00904.x)

[118] Parra G, Schick R, Corkeron P 2006 Spatial distribution and environmental correlates of Australian snubfin and Indo-Pacific humpback dolphins. Ecography 29, 396–406. (doi:10.1111/j.2006.0906-7590.04411.x)

[119] Neumann DR 2001 Seasonal movements of short-beaked common dolphins (*Delphinus delphis*) in the north-western Bay of Plenty, New Zealand: influence of sea surface temperature and El Nino/La Nina. N. Z. J. Mar. Freshw. Res. 35, 371–374. (doi:10.1080/00288330.2001.9517007)

[120] The State of Victoria. 1995 Fisheries Act 1995.

[121] The State of Victoria. 2016 Fisheries (Catch Limit) Amendment Regulations 2016.

[122] Lusseau D 2003 Effects of tour boats on the behavior of bottlenose dolphins: using Markov chains to model anthropogenic impacts. Conserv. Biol. 17, 1785–1793. (doi:10.1111/j.1523-1739.2003.00054.x)

[123] Constantine R, Brunton DH, Dennis T 2004 Dolphin-watching tour boats change bottlenose dolphin (*Tursiops truncatus*) behaviour. Biol. Conserv. 117, 299–307. (doi:10.1016/j.biocon.2003.12.009)

[124] Neumann DR, Orams MB 2005 Behaviour and ecology of common dolphins (*Delphinus delphis*) and the impact of tourism in Mercury Bay, North Island, New Zealand. Wellington, New Zealand: Department of Conservation.

[125] Stockin KA, Lusseau D, Binedell V, Wisemen N, Orams MB 2008 Tourism affect the behaviour budgets of the common dolphin *Delphinus sp*. in the Hauraki Gulf, New Zealand. Mar. Ecol. Progr. Press 355, 287–295. (doi:10.3354/meps07386)

[126] Meissner AM, Christiansen F, Martinez E, Pawley MD, Orams MB, Stockin KA 2015 Behavioural effects of tourism on oceanic common dolphins, *Delphinus* sp., in New Zealand: the effects of Markov analysis variations and current tour operator compliance with regulations. PLoS ONE 10, e0116962 (doi:10.1371/journal.pone.0116962) 25565523 10.1371/journal.pone.0116962 PMC4286237

[127] The State of Victoria. 2009 Wildlife (Marine Mammals) Regulations.

[128] Mason S, Salgado Kent C, Donnelly D, Weir J, Bilgmann K 2016 Data from: Atypical residency of short-beaked common dolphins (*Delphinus delphis*) to a shallow, urbanized embayment in south-eastern Australia. Dryad Digital Repository. (doi:10.5061/dryad.5j1k1) 10.1098/rsos.160478 PMC5043329 27703709

